# Malalignment of the total ankle replacement increases peak contact stresses on the bone-implant interface: a finite element analysis

**DOI:** 10.1186/s12891-022-05428-0

**Published:** 2022-05-17

**Authors:** Sanne W. G. van Hoogstraten, Joris Hermus, Arjan C. Y. Loenen, Jacobus J. Arts, Bert van Rietbergen

**Affiliations:** 1 Department of Orthopedic Surgery, Laboratory for Experimental Orthopedics, Maastricht University Medical Center, Maastricht, the Netherlands; 2 Department of Biomedical Engineering, Orthopedic Biomechanics, Eindhoven University of Technology, Eindhoven, the Netherlands

**Keywords:** Total ankle replacement, Malalignment, Finite element modeling, Contact stress, Bone-implant interface

## Abstract

**Introduction:**

Malalignment of the Total Ankle Replacement (TAR) has often been postulated as the main reason for the high incidence of TAR failure. As the ankle joint has a small contact area, stresses are typically high, and malalignment may lead to non-homogeneous stress distributions, including stress peaks that may initiate failure. This study aims to elucidate the effect of TAR malalignment on the contact stresses on the bone-implant interface, thereby gaining more understanding of the potential role of malalignment in TAR failure.

**Methods:**

Finite Element (FE) models of the neutrally aligned as well as malaligned CCI (Ceramic Coated Implant) Evolution TAR implant (Van Straten Medical) were developed. The CCI components were virtually inserted in a generic three-dimensional (3D) reconstruction of the tibia and talus. The tibial and talar TAR components were placed in neutral alignment and in 5° and 10° varus, valgus, anterior and posterior malalignment. Loading conditions of the terminal stance phase of the gait cycle were applied. Peak contact pressure and shear stress at the bone-implant interface were simulated and stress distributions on the bone-implant interface were visualized.

**Results:**

In the neutral position, a peak contact pressure and shear stress of respectively 98.4 MPa and 31.9 MPa were found on the tibial bone-implant interface. For the talar bone-implant interface, this was respectively 68.2 MPa and 39.0 MPa. TAR malalignment increases peak contact pressure and shear stress on the bone-implant interface. The highest peak contact pressure of 177 MPa was found for the 10° valgus malaligned tibial component, and the highest shear stress of 98.5 MPa was found for the 10° posterior malaligned talar model. High contact stresses were mainly located at the edges of the bone-implant interface and the fixation pegs of the talar component.

**Conclusions:**

The current study demonstrates that TAR malalignment leads to increased peak stresses. High peak stresses could contribute to bone damage and subsequently reduced implant fixation, micromotion, and loosening. Further research is needed to investigate the relationship between increased contact stresses at the bone-implant interface and TAR failure.

## Introduction

An increasingly used treatment for end-stage ankle osteoarthritis is total ankle replacement (TAR). With TAR, a prosthetic implant between the tibia and talus replaces the ankle joint, maintaining tibiotalar articulation. Over the past decade, TAR has been increasingly used in the clinic and has challenged ankle arthrodesis as the treatment of choice for end-stage ankle osteoarthritis as patient satisfaction, pain relief, and ankle function continued improving [1]. Unfortunately, primary concerns for TAR are still present including longevity and rate of revision. A study by Spirt et al. showed that 28% of the patients that received a total ankle arthroplasty, underwent at least one reoperation due to complications [2]. Subsidence and aseptic loosening are the most common clinical reasons for TAR failure, as they occur in 10.7 and 8.7% of all patients with a TAR [3]. TAR has unsatisfying long-term outcomes, with a survival rate of 70% after 10 years and less than 50% after 14 years [4–6]. Despite the development and improvement of four generations of TAR designs, the potential risk of persisting pain and low functional outcome after TAR surgery remains high.

Malalignment has been often postulated as one of the main reasons for the high failure rate of TAR. The surgical procedure of a TAR is challenging, and it is especially complicated to achieve the correct alignment of the TAR components. Also, the survival rate of the TAR increases significantly with increasing surgical experience, showing the presence of a significant learning curve [7, 8]. Furthermore, restoring alignment in patients with a pre-operative deformity of the ankle is even more challenging. Proper alignment is essential for a successful TAR surgery as a slight degree of malalignment has been claimed to result in higher failure rates [9–12].

The ankle joint has a contact area approximately three times smaller than that of the hip or knee joint, but it experiences higher forces [13]. Peak forces of 2.5-, 4-, and 6-times body weight were found for respectively the knee, hip, and ankle [14]. Together, the small contact area and large forces result in high contact stresses in the ankle joint [14]. The implant material, often cobalt-chrome-molybdenum (Co-Cr-Mo) alloys, is substantially stiffer than cortical and trabecular bone, so transmission of force from the implant to the bone can give stress peaks on the bone-implant interface [15]. High stress peaks can contribute to bone damage and subsequently reduced implant fixation, micromotion, and loosening [16].

In several studies, Finite Element (FE) modeling has been used to investigate the biomechanical consequences of TAR malalignment and the role of these biomechanical parameters in TAR failure [17–21]. FE models of different TAR designs have been developed and showed that TAR malalignment can result in increased contact pressure on the polyethylene liner, leading to wear particles and implant loosening [21]. It was also found that malalignment increases micromotion, which can lead to improper fixation of the TAR [20]. Furthermore, tibial bone strains alter upon malalignment of the TAR, leading to local overloading and stress shielding which can contribute to implant loosening [18].

The effect of TAR malalignment on the contact stresses on the bone-implant interface, however, has not been investigated using FE modeling. Therefore, it remains unclear to what extent malalignment of the TAR affects the stresses at the bone-implant interface. Gaining insight into the contact stresses on the bone-implant interface in neutrally and malaligned TARs might lead to a better understanding of the high failure rate of TAR. Therefore, this study aims to elucidate the effect of TAR malalignment on the contact stresses on the bone-implant interface of a TAR, by developing a generic FE model of the neutrally and malaligned TAR and calculating the resulting contact stresses under physiological loading conditions.

## Methods

### Geometrical reconstructions

**Fig. 1:**
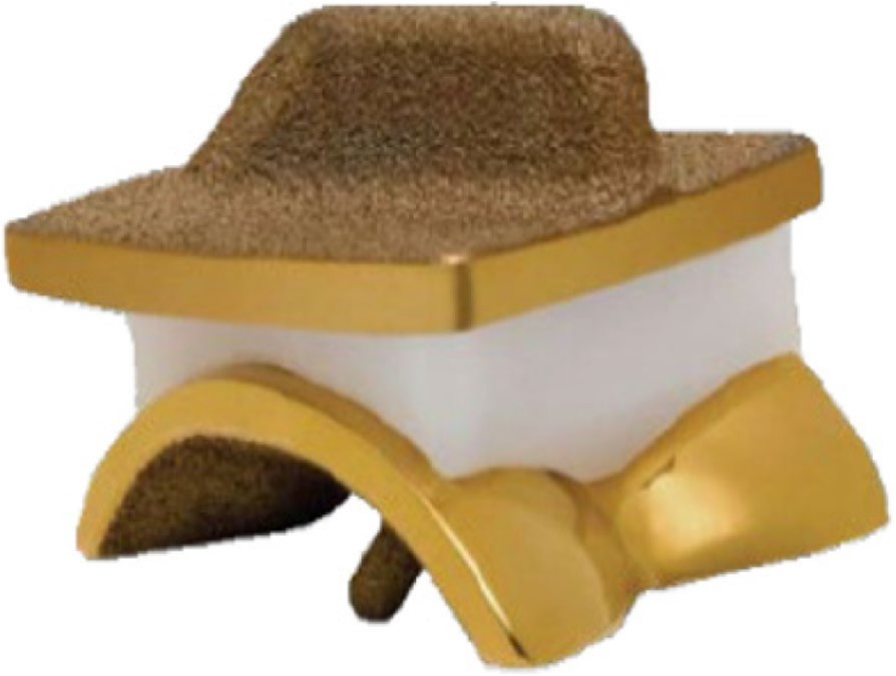
The CCI Evolution TAR implant (Van Straten Medical). The three components from top to bottom: tibial CoCrMo component, polyethylene liner, talar CoCrMo component

FE models of the neutrally aligned and malaligned CCI Evolution TAR [22] (Van Straten Medical, The Netherlands; Fig. 1) were created, by virtually inserting the tibial and talar CCI component in respectively a tibia and talus. The CCI Evolution TAR consists of a tibial and talar cobalt-chrome-molybdenum (Co-Cr-Mo) component and a polyethylene liner (Fig. 1). The Co-Cr-Mo components in contact with the bone were included in this study and the geometries of the standard-sized tibial and talar components of the CCI Evolution TAR design were obtained by three-dimensional (3D) scanning using the ATOS Scanport (Zebicon a/s; Billund). From 2010 to 2016, 65 CCI Evolution TAR implants were placed at Maastricht University Medical Centre. A pre-operative CT scan with a slice thickness of 0.6 mm of a randomly selected, anonymized patient from this cohort (male, age 72 years) with a clinically well-performing TAR was used. The CT scan was obtained during clinical follow-up in the past and no additional interventions or radiographical assessments were needed for this retrospective study. This CT scan was used for virtual reconstruction of the tibia and talus. Using medical image processing software (MIMICS® version 21.0; Materialise NV, Leuven, Belgium), the tibia and talus of the left foot were segmented and 3D reconstructions were obtained. In MIMICS, the CCI components were virtually inserted in the tibia and talus, following surgical guidelines and with guidance from an orthopedic surgeon specialized in TAR surgery. The CCI was placed in neutral alignment, which is defined as perpendicular to the anatomical axis of the tibia measured from the tibial TAR plateau.

**Fig. 2:**
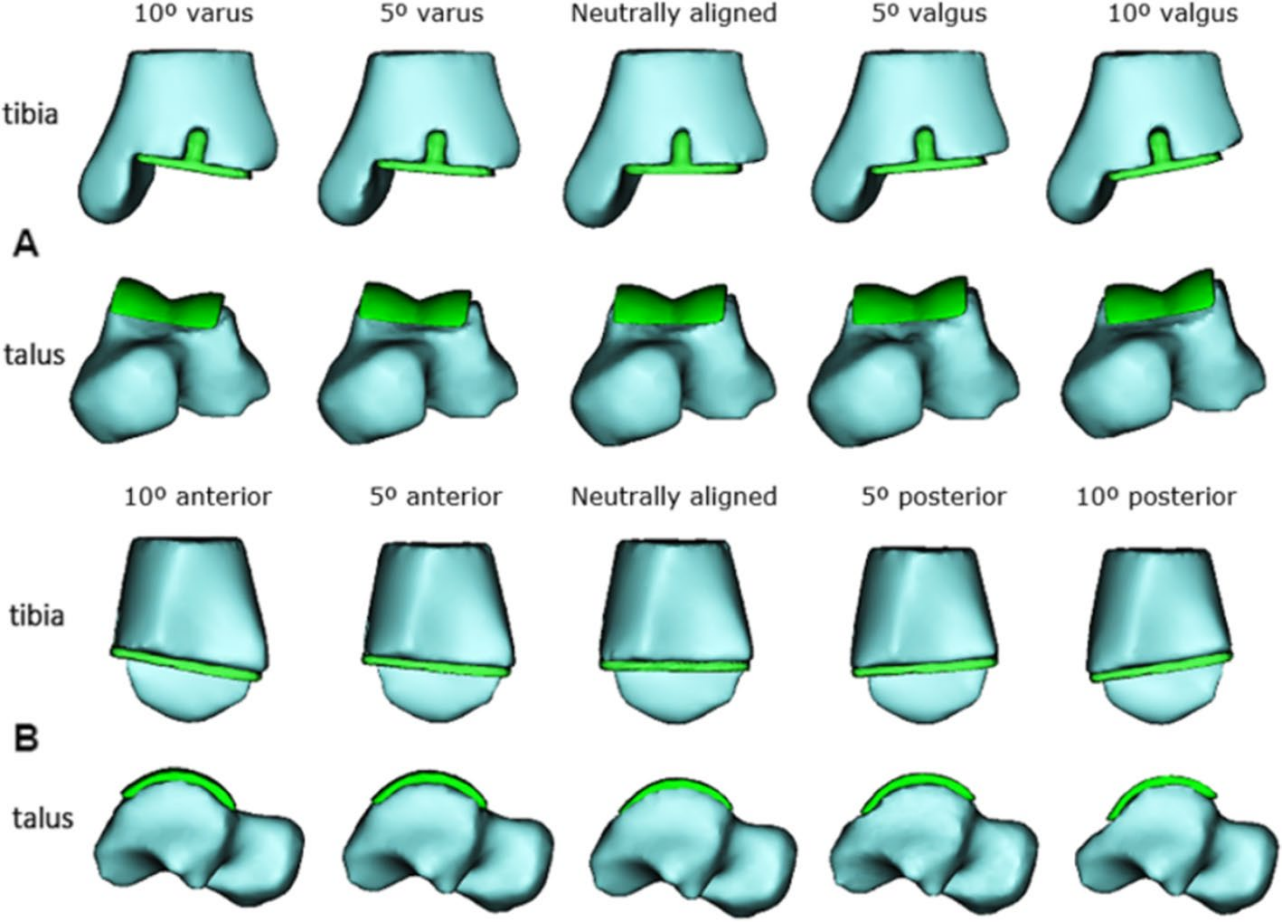
Coronal and sagittal (mal) alignment of the CCI Evolution TAR

Besides the neutrally aligned implant, reconstructions of the malaligned TAR components were created. The tibial and talar TAR components in 5° and 10° varus, valgus, anterior, and posterior malalignment were modeled, as can be seen in Fig. 2. These are the malalignment cases measured and observed in the clinic [20, 21, 23, 24]. For the anterior-posterior malaligned implants, the sagittal angle deviated from the neutrally aligned CCI, where the anteriorly malaligned implant is in plantarflexion relatively to the foot and the opposite for the posterior malaligned implant. In total, 9 reconstructions were developed for both the tibial and talar component, resulting in a total of 18 models.

### Material properties

All the materials were modeled as homogenous, isotropic, and linear elastic, with material properties as reported in Table 1. The cortical shell and the trabecular bone of the tibia and talus were assigned separate material properties. For the genericity of the model, the trabecular bone was modeled as a continuum.

**Table 1:** Material properties

	Young’s Modulus [MPa]	Poisson’s ratio [−]	
Co-Cr-Mo [25, 26]	210·10^3^	0.29	
Cortical bone [27, 28]	17·10^3^	0.3	
Trabecular bone [29, 30]	500	0.3	

### Contact

To model the direct post-operative interaction after TAR surgery, a contact condition with a coefficient of friction of 0.5 at the bone-implant interface was chosen [31]. ‘Hard’ linear contact with a penalty method and automatically calculated contact stiffness was used to simulate the contact behavior in the normal direction at the bone-implant interface. Small-sliding formulation with the surface-to-surface discretization method was used.

### Boundary conditions and meshing

**Fig. 3:**
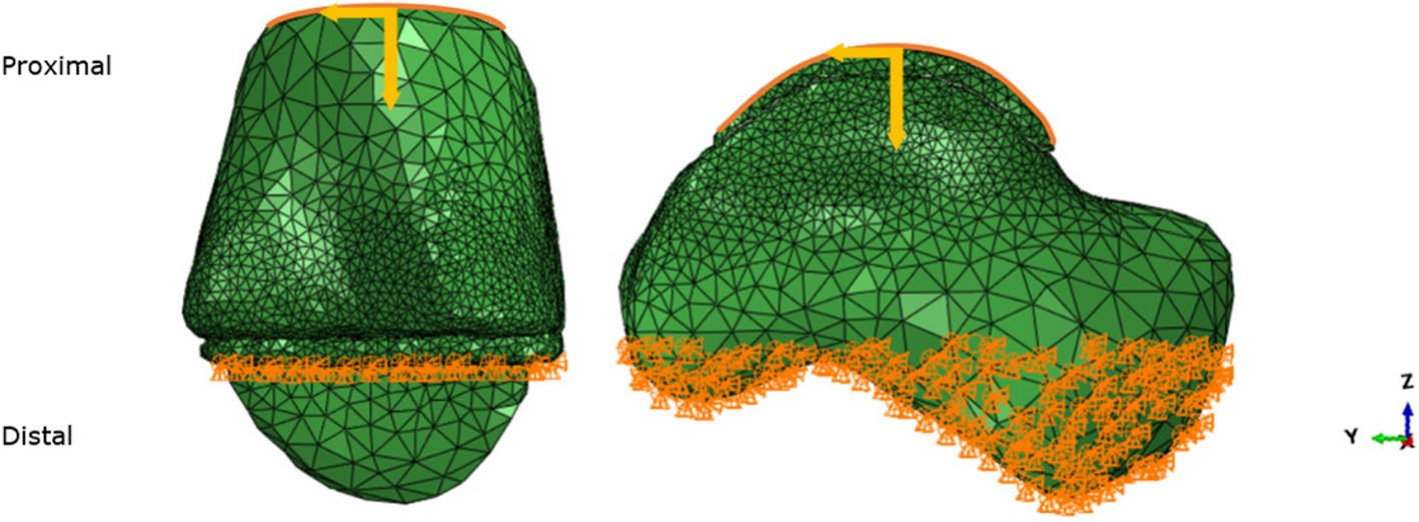
Boundary and loading conditions of the neutral aligned tibial (left) and talar (right) TAR models. Load distributed along surface indicated by the orange line. The yellow arrows indicate the loading components

For the tibial models, the bottom of the distal plateau of the tibial TAR component was constrained in all directions. For the talar models, the distal part of the talus was fixed to all motions (Fig. 3). Using medical image processing software (3-Matic® version 16.0; Materialise NV, Leuven, Belgium), automatic meshing was performed using 10 node tetrahedral elements (C3D10). A maximum triangle edge length of 5 mm was chosen, with a denser meshing near the bone-implant interface using a maximum edge length of 1.5 mm. This edge length was assumed acceptable based on previous models [20, 32]. The models consisted of 55,000 to 64,000 nodes.

### Loading

Loading was applied as a point force evenly distributed between the nodes of the proximal tibia and the proximal surface of the talar TAR component, for respectively the tibial and talar models. For all models, the bone-implant interface stresses were investigated during the terminal stance, when the ankle joint is subjected to the highest axial reaction force during a normal gait cycle (5.2 times bodyweight). A bodyweight of 82 kg was assumed (corresponding to the patient from the CT scan) so an axial load of − 4183 N was applied through the tibial axis (z-axis). During terminal stance, however, bodyweight is not transmitted through the tibial axis but at a 15° angle in anterior direction from the tibial axis meaning a shear component is present. Therefore, a shear load of 1121 N was applied in the direction of the tibial plateau (y-axis) [19, 33–35].

### Numerical method and outcome measures

**Fig. 4:**
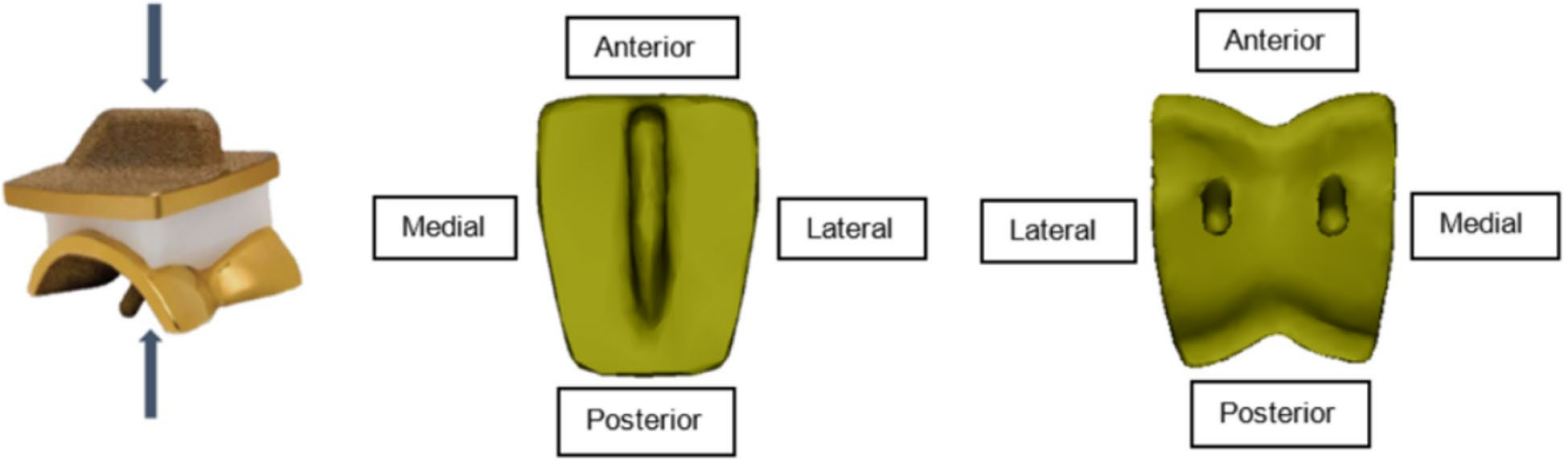
Views of visualization for the stress distribution images

The models were processed using Abaqus® Standard/Implicit FE solver (ABAQUS CAE, ver. 2019, SIMULIA, Providence, RI, USA). The models had a runtime of approximately 21 hours, using a computer with a CPU Intel core Xeon X5550 2.6 GHz 4-cores and 20 GB RAM. A customized MATLAB® script (version 2020, MathWorks Inc., Natick, MA, USA) was coded to extract output parameters from the output file from Abaqus and to obtain the peak contact stress on the bone-implant interface. Contact pressure and shear stress were obtained (CPRESS and CSHEAR in Abaqus). For the models showing the most indicative quantitative results, stress distribution plots were made. These images were created by plotting the bone-implant interface contact stresses on the TAR surfaces, as shown in Fig. 4.

## Results

### Malalignment of the tibial TAR component

**Fig. 5:**
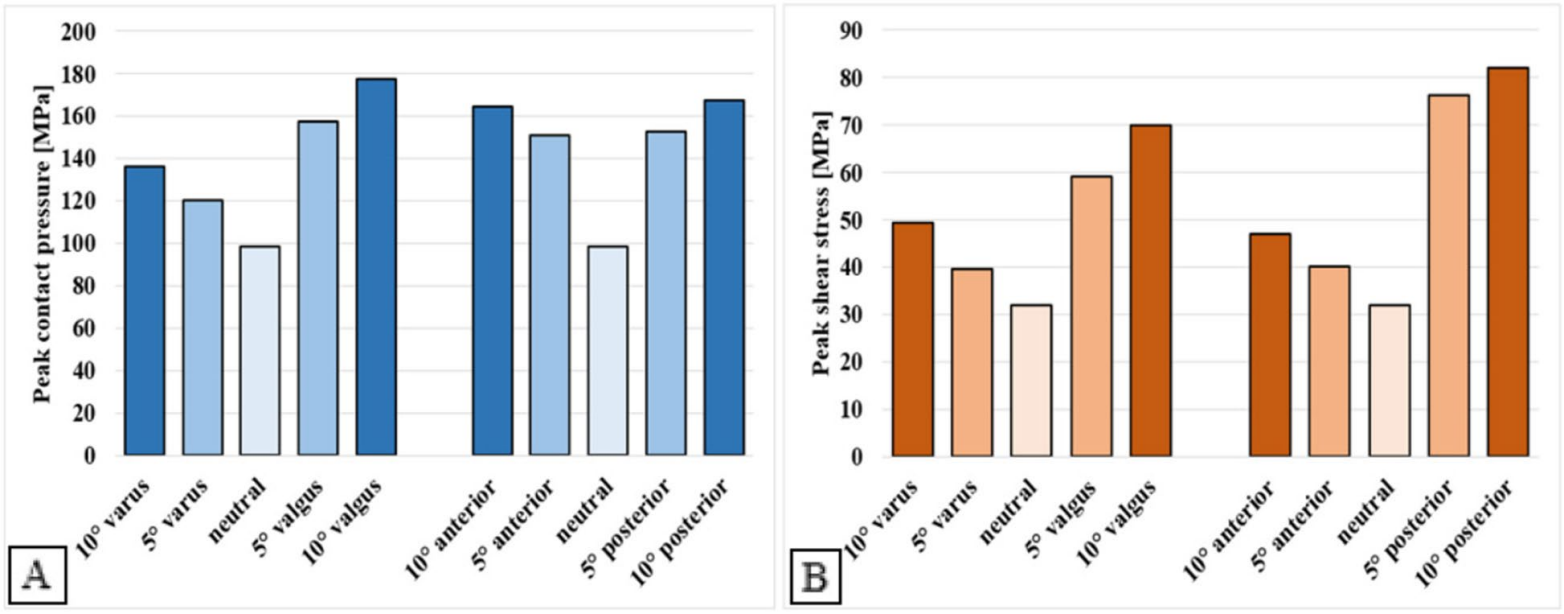
Peak contact pressure (**A**) and shear stress (**B**) for the neutrally aligned and malaligned tibial TAR components

**Fig. 6:**
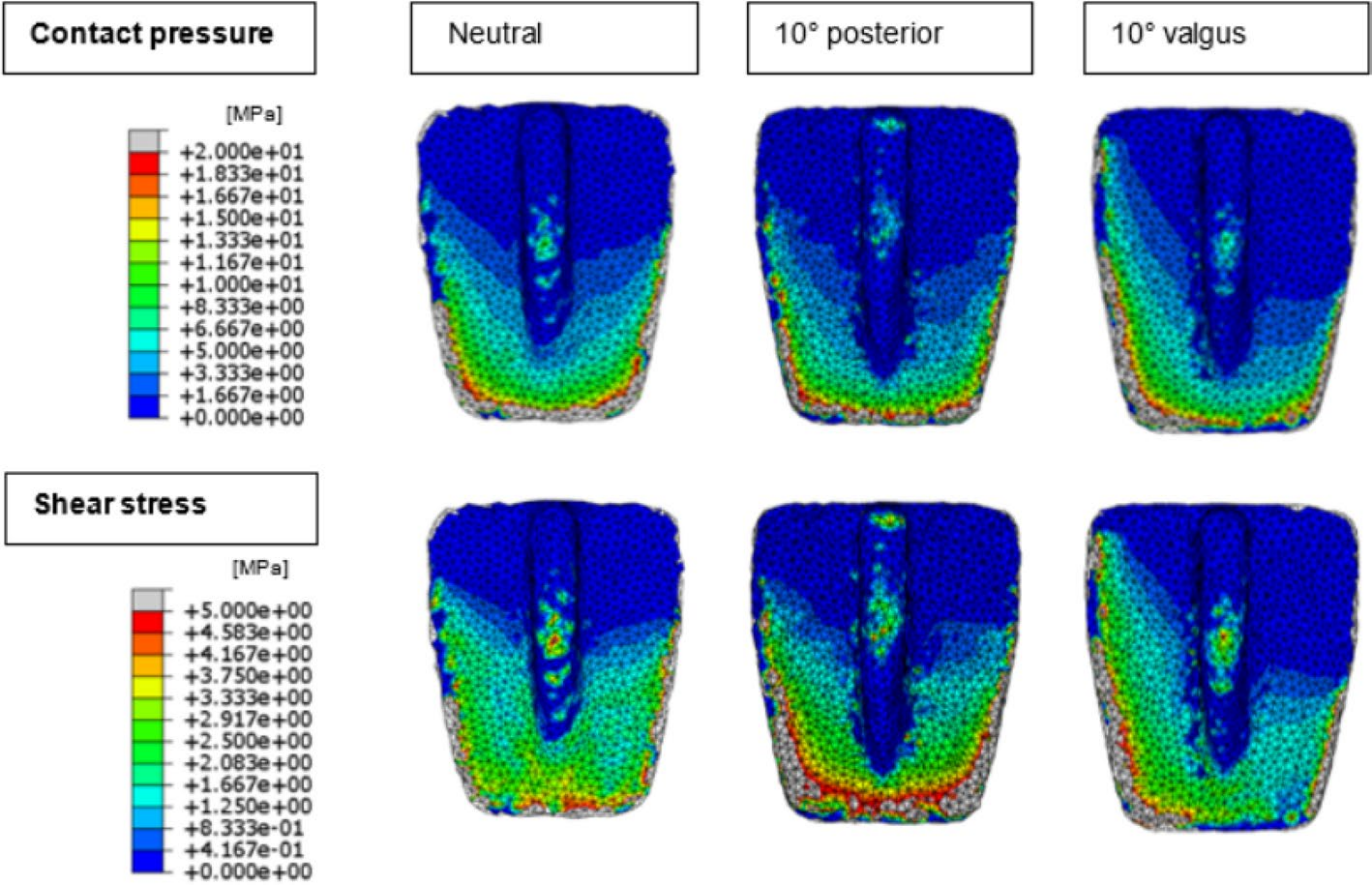
Contact pressure and shear stress distribution images of the neutral, 10° posterior, and 10° valgus malaligned tibial models

In Fig. 5, peak contact pressures and shear stresses of the malaligned and neutrally aligned tibial TAR models are plotted. For the neutrally aligned tibial TAR component, a peak contact pressure of 98.4 MPa and a peak shear stress of 31.9 MPa is found. Peak contact stresses increase upon TAR malalignment, where valgus and posterior malalignment show the largest increases. A maximum peak contact pressure of 177 MPa was found for the 10° valgus malaligned tibial component and the highest shear stress found was 82.2 MPa for the 10° posterior malaligned tibial model. For both the tibial and talar models, stress distribution plots were made for the 10° posterior and valgus malaligned models, as the quantitative results of these models provide the most insights. Clear changes in shear stress distribution on the tibial interface are visible upon malalignment, as can be seen in Fig. 6. Shear stresses expectedly shift towards the medial and posterior side of the interface for respectively the valgus and posterior malaligned models.

### Malalignment of the talar TAR component

**Fig. 7:**
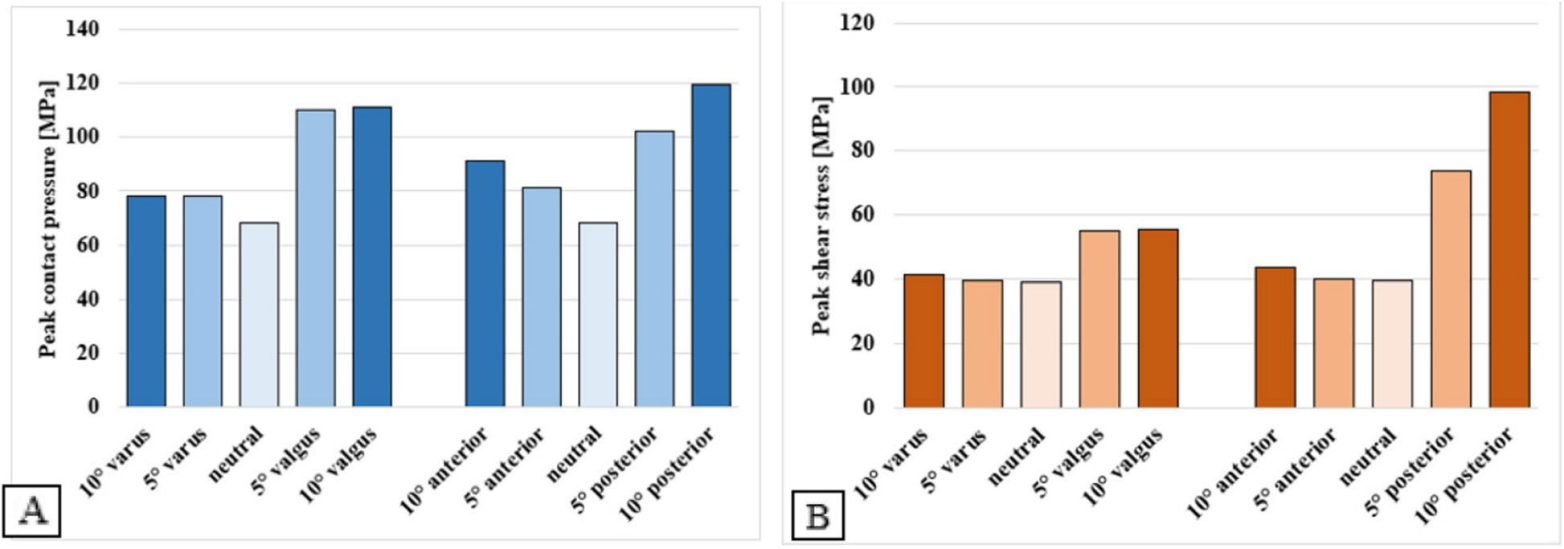
Peak contact pressure (**A**) and shear stress (**B**) for the neutrally aligned and malaligned talar TAR components

**Fig. 8:**
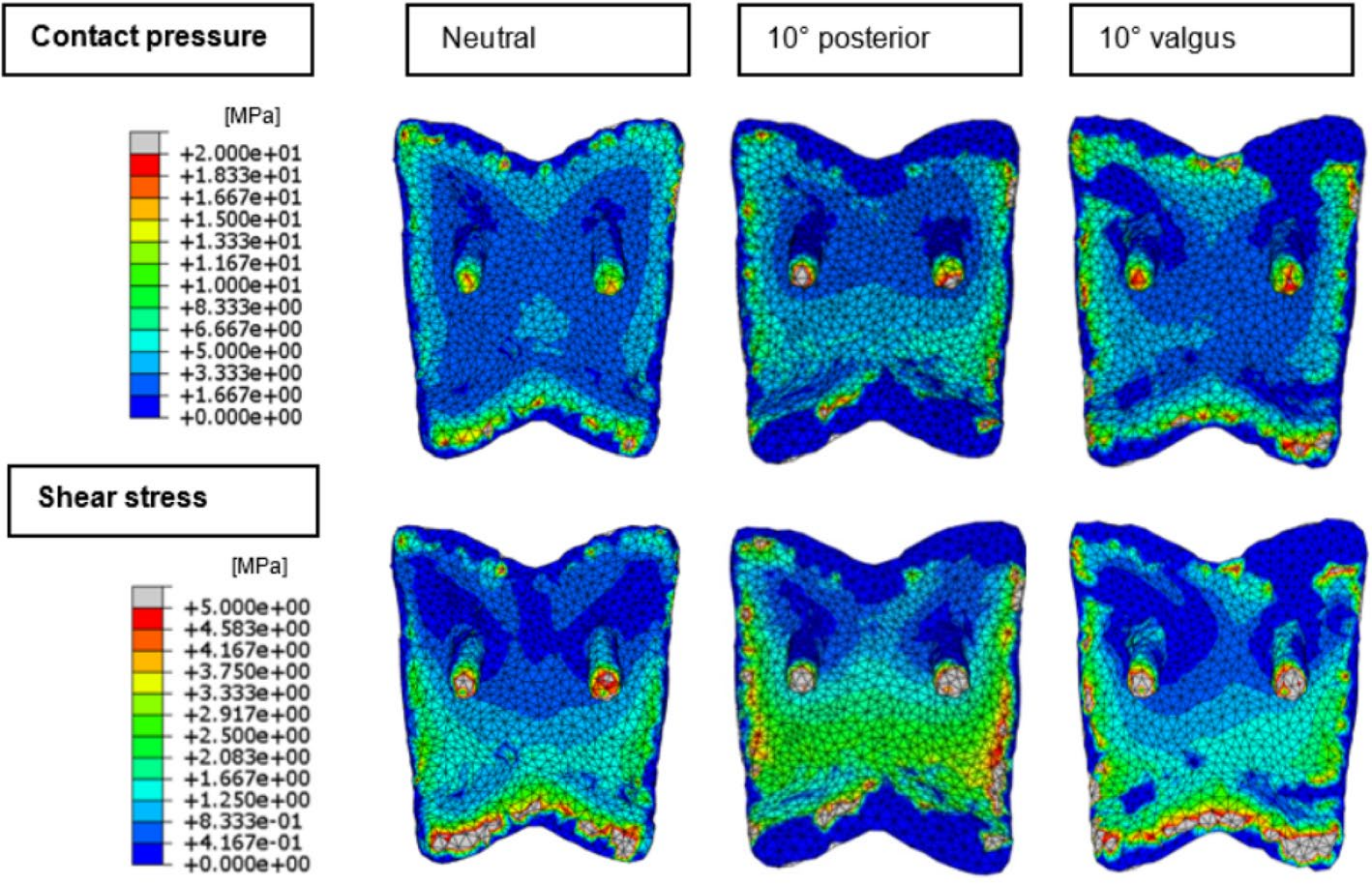
Contact pressure and shear stress distribution images of the neutral, 10° posterior, and 10° valgus malaligned talar models

Peak contact pressures and shear stresses on the talar bone-implant interface are plotted in Fig. 7. In neutral alignment, a peak contact pressure of 68.2 MPa and a peak shear stress of 39.0 MPa were found. As with the tibial models, peak contact stresses increase upon TAR malalignment. Also corresponding to the tibial models, valgus and posterior malaligned TAR components showed the largest increase in contact stress. Both maximum peak contact pressure and shear stress were found for the 10° posterior model of respectively 120 MPa and 98.5 MPa. Peak shear stresses on the talar bone-implant interface show fewer changes upon TAR malalignment than observed for the tibial interface, except for the posteriorly malaligned models, where a large increase was seen in peak shear stress. Changes in stress distributions are visible in Fig. 8. Expected shifts in shear stress distribution towards the lateral and posterior side of the bone-implant interface are visible on the talar bone-implant interface, upon respectively valgus and posterior TAR malalignment. Contact pressure distributions show less pronounced changes upon TAR malalignment, compared to the tibial models.

## Discussion

The purpose of this study was to elucidate the effect of TAR malalignment on the contact stresses on the tibial and talar bone-implant interface. The results of this study show that TAR malalignment can substantially increase local peak stresses (by up to 158%) on the bone-implant interface and that proper positioning of the TAR thus is necessary to reduce contact stresses on the bone-implant interface. Peak contact pressures up to 177 MPa were found which are exceeding the ultimate yield point of trabecular bone, and thus presumably leads to bone damage [36]. Bone damage, in turn, can lead to implant loosening, subsidence, and subsequent TAR failure. Even though the generic character of the presented models cannot lead to clinical recommendations, it was shown that contact pressure on the bone-implant interface can be dangerously high and that contact stresses on the bone-implant interface are important parameters to include in future FE studies which evaluate the correlation between biomechanical load on the construct and the eventual clinical TAR performance.

The neutrally aligned TAR components showed lower contact stresses than the malaligned models. Similar results, although for other output parameters, were obtained by other FE studies investigating the biomechanical consequences of TAR malalignment for other TAR designs [18, 20, 21]. Contact pressure on the polyethylene liner, micromotion, and the occurrence of strain shielding are lower when the TAR is placed in neutral alignment. In previous research, however, it was shown that large variation in results can be found when investigating different TAR designs [20, 32]. As every study models different implant types with varying loading regimes and boundary conditions, it is not trivial to make a direct comparison with previous literature.

The stress distribution images show that in neutrally aligned as well as malaligned TARs, contact pressures, and shear stresses are unequally divided over the bone-implant interface during terminal stance and that high stresses are present on the edges of the bone-implant interface and on the talar fixation pegs. Uneven distribution of loads can contribute to component subsidence, one of the most common complications of TAR failure, due to local overloading [37, 38]. Furthermore, the high stress peaks located on the talar fixation pegs, which serve as the anchor of the talar component resisting it from rotating on the talar surface, might result in fixation problems and subsidence of the talar component. Valgus and posterior malalignment of the CCI Evolution TAR components showed the largest increase in contact stress on the bone-implant interface, higher than varus and anterior malalignment. Also, the tibial TAR component showed higher contact pressure peaks than the talar component. This is in accordance with Sopher et al., which reported higher micromotion and strain outputs for the tibial component than for the talar component [20]. Besides the cases of malalignment modeled in the current study, Sopher et al. showed that a malalignment with a gap between the implant and bone led to high micromotions. Therefore, in future studies, it might be interesting to assess the effect of a gap between the implant and the bone on the bone-implant interface stresses, as focal loading is expected due to a decrease in the contact area of the implant.

A main limitation of the presented study is the lack of experimental validation. A cadaver study of the differently aligned CCI Evolution TAR using for example the K-scan Joint Analysis System (TekScan Inc., Boston, MA), could be of great value and should be included in a future validation study. Some other limitations must be highlighted as well. Soft tissues, such as ligaments, were not considered in the model, but as these do not carry much load during the loading conditions applied in the present model it is expected that this omission does not affect the results. Furthermore, the polyethylene liner was not taken into account in the presented FE models. Loading on the talar TAR component was applied by distributing a point force on the proximal talar TAR component surface, but loads will actually be transmitted through the moving polyethylene liner located in between the tibial and talar component. This liner has a small surface, so stress distributions at the implant surface are expected to be more focused. Nevertheless, as the implant is very stiff compared to the bone, this should not affect the results at the bone-implant interface. Furthermore, we only analyzed the direct post-operative case, where no bonding between bone and implant has taken place. After such bonding occurs, the stresses may be reduced. The results presented here are thus more representative for early failure. Also, the amount of elements was limited due to computational resources and number of model variants. This limitation, however, was assumed acceptable based on previous models and the use of consistent element size throughout all model variants [20, 32]. Lastly, we focused solely on the terminal stance phase of the gait cycle since the highest axial load is present during this phase. The highest peak contact pressures are expected during this phase of the gait cycle, but analysis of the complete gait cycle might further elucidate the effect of TAR malalignment on the overall stress distributions on the bone-implant interface.

## Conclusions

In conclusion, the presented results show that TAR malalignment leads to a considerable increase in peak contact stresses on the bone-implant interface, which may lead to bone damage and subsequent TAR loosening or subsidence. It was found that valgus and posterior malalignments induce the largest increase in peak stress for the CCI Evolution TAR design. We further elucidated the possible failure mechanism of the CCI Evolution TAR, and this study showed that contact stresses on the bone-implant interface are an important parameter to include when investigating TAR malalignment and performance.

## Data Availability

All data generated and analyzed during this study are included in this published article or depicted in figures in this published article.

## Abbreviations

TAR: Total Ankle Replacement
FE: Finite Element
CT: Computed Tomography
3D: three-dimensional
2D: two-dimensional
CCI: Ceramic Coated Implant
Co-Cr-Mo: Cobalt-Chrome-Molybdenum
